# Research progress on the mechanism of exosome-mediated virus infection

**DOI:** 10.3389/fcimb.2024.1418168

**Published:** 2024-06-26

**Authors:** Hanjia Zhang, Xuanyi Liu, Jiuming Shi, Xuan Su, Jiayuan Xie, Qingfeng Meng, Hao Dong

**Affiliations:** ^1^ College of Life Sciences, Jilin Agricultural University, Changchun, Jilin, China; ^2^ Engineering Research Center of Bioreactor and Pharmaceutical Development, Jilin Agricultural University, Changchun, China

**Keywords:** exosomes, virus infection, mechanism, microRNAs (miRNAs), endosomal sorting complex required for transport (ESCRT)

## Abstract

Exosomes are extracelluar vesicles that facilitate intercellular communication and are pivotal in post-transcriptional regulation within cellular gene regulatory networks, impacting pathogen dynamics. These vesicles serve as crucial regulators of immune responses, mediating cellular interactions and enabling the introduction of viral pathogenic regions into host cells. Exosomes released from virus-infected cells harbor diverse microRNAs (miRNAs), which can be transferred to recipient cells, thereby modulating virus infection. This transfer is a critical element in the molecular interplay mediated by exosomes. Additionally, the endosomal sorting complex required for transport (ESCRT) within exosomes plays a vital role in virus infection, with ESCRT components binding to viral proteins to facilitate virus budding. This review elucidates the roles of exosomes and their constituents in the invasion of host cells by viruses, aiming to shed new light on the regulation of viral transmission via exosomes.

## 1 Introduction

### 1.1 Introduction of exosomes

Exosomes are a type of extracellular vesicles (EVs) secreted by cells, which have a lipid bilayer membrane with a diameter of 30nm~150nm (Marote et al., 2016), a buoyancy density of 1.13~1.19g/mL in a sucrose gradient (Yellon and Davidson, 2014), and a cup-shaped vesicle structure under an electron microscope (Zakharova et al., 2007). Exosomes are widely present in various biological fluids, and all cells can secrete exosomes. In 1983, Pan et al. (Pan and Johnstone, 1983) first discovered a small vesicle capable of transporting the transferrin receptor to the extracellular space during the maturation of sheep reticulocytes, and it was named “Exosome” by Johnstone in 1987 (Johnstone et al., 1987). Initially considered merely a means of discarding metabolic waste and removing obsolete membrane proteins during erythrocyte maturation, exosomes are now recognized as “communicators” between cells. They carry signaling molecules that interact with target cells, influencing their physiological and pathological states, and are intimately connected with disease processes and treatments (Kalluri, 2016).

### 1.2 The composition of exosomes

**Figure 1:**
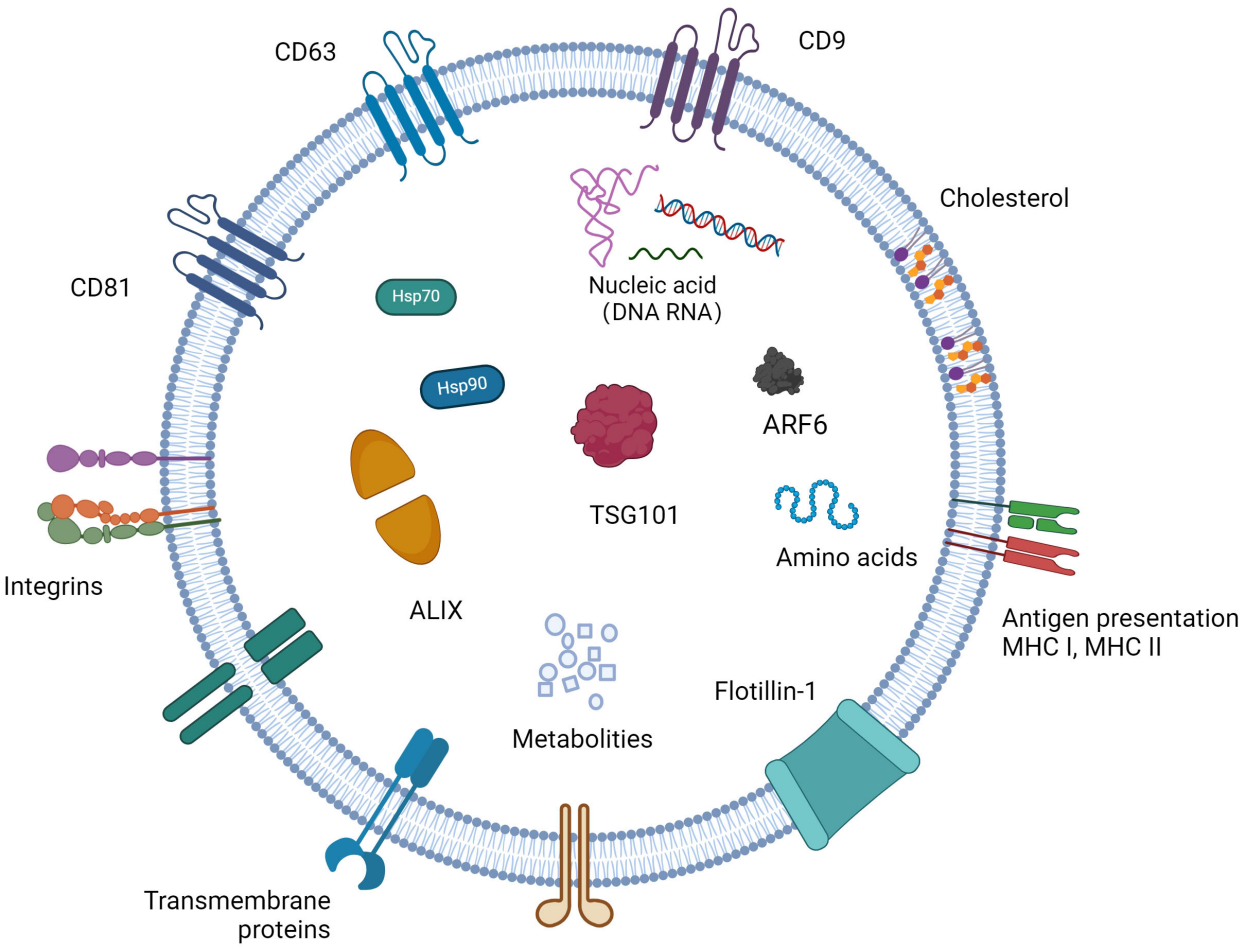
The content of exosomes and biomarkers for exosomes. Exosomes have a phospholipid bilayer, which has the same topology as cells, and their bilayer membrane contains sphingomyelin, phosphatidylserine, cholesterol, ceramide, etc., which protects their inclusions from degradation. Exosomes contain a variety of bioactive substances, mainly composed of proteins, nucleic acids and lipids. Different cells carry different bioactive ingredients in different states.

Exosomes consist of functional proteinss, lipids, and nucleic acids (miRNAs, lncRNAs, circRNAs, rRNAs, etc.) (Zhou S. et al., 2022). The RNA in the exosome is transferred from the parent cell to the receptor cell. The protein composition of exosomes varies depending on the cells that secrete them, and their surface features a range of biomarkers, such as the tetraspanins CD9, CD63, CD81, CD82, heat shock protein HSP70, HSP90, the MVB-forming proteins TSG101, ALIX, etc. (**Figure 1**) (Nam et al., 2020). Beyond these common proteins, exosomes contain a variety of specific proteins that change according to their physiological and pathological conditions. Furthermore, exosomes are enriched with cholesterol, sphingomyelin, sphingosine, phosphatidylserine, and ceramide (Li S. et al., 2021), crucial for maintaining exosomal structure, biogenesis, and regulating the homeostasis of recipient cells.

### 1.3 Biogenesis of exosomes

**Figure 2:**
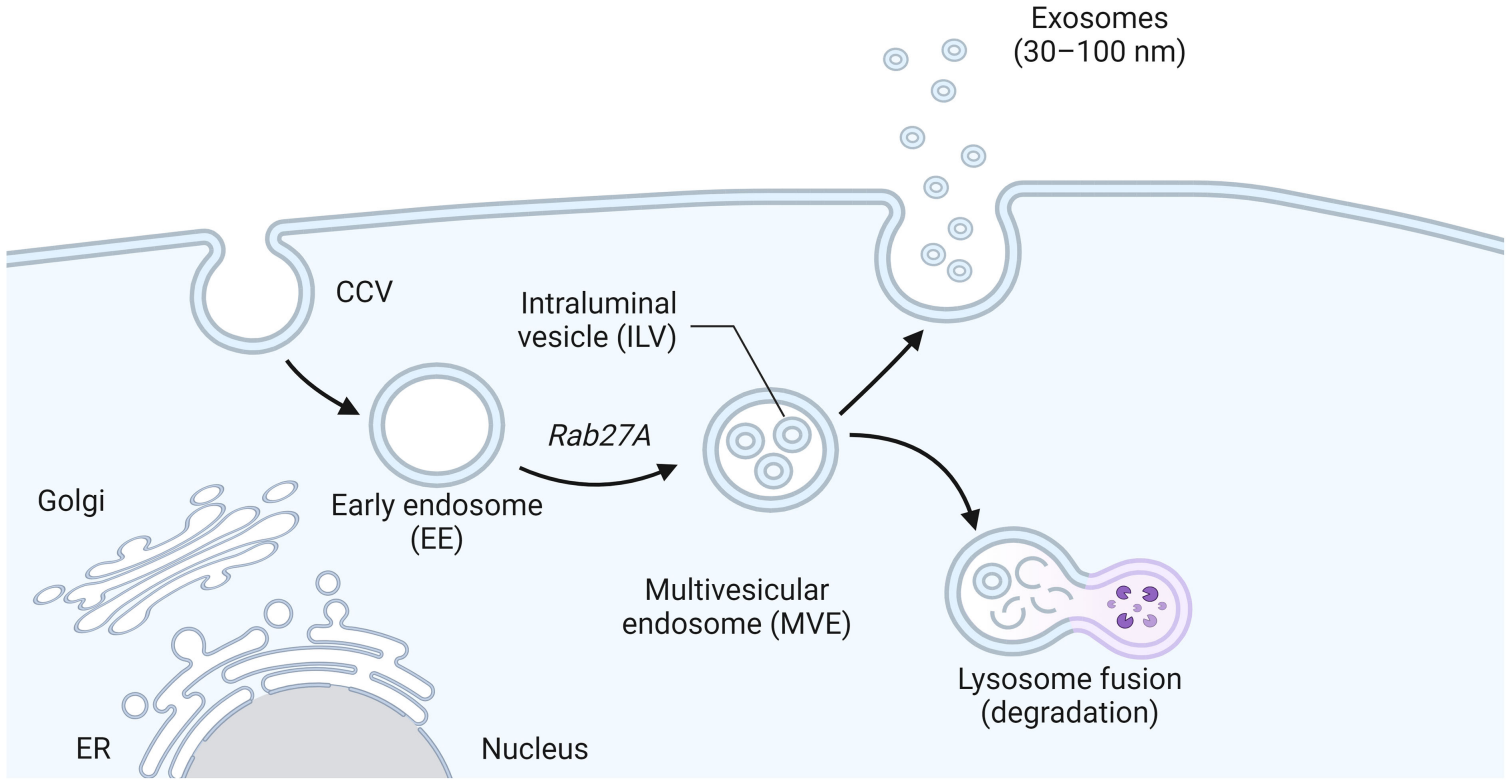
The biogenesis of exosomes. Early endosomes formed by plasma membrane invagination grew into late endosomes and MVB containing intracavitary vesicles (ILVs) after the formation of ILV. Part of MVBs fuses with the cell membrane and is released into the extracellular space as exosomes, while the other part is degraded by lysosomes.

Exosomes originate from multivesicular bodies (MVBs), where the plasma membrane inwardly sprouts to form the early endosomes, which mature into the late endosomes and MVBs containing Intraluminal vesicles (ILVs), after the formation of ILVs (Samuels et al., 2023). A part of MVBs is released into the extracellular space as exosomes after fusion with the cell membrane, while another part is degraded by lysosomes (**Figure 2**) (Marote et al., 2016). The exosomal secretion process requires the aid of the endosomal sorting complex required for transport (ESCRT), a family of proteins that includes four complexes: ESCRT-0, ESCRT-I, ESCRT-II, and ESCRT-III (Addi et al., 2020). Escrt-0 regulates content aggregation through a ubiquitination-dependent pathway, ESCRT-I and ESCRT-II induce bud formation, ESCRT-III drives vesicle shedding, and helper proteins (VPS4 ATPase) mediate dissociation and circulation of the ESCRT system. ESCRT-0: The ESCRT-0 complex (HRS, STAM) is not involved in the process of budding and membrane separation, so it can be used as a basis to trace the origin of exosomes (Duguez, 2021). HRS recognizes monoubiquitinated proteins and binds to the ubiquitination site to target the protein to ILVs (the process of protein sorting into ILVs is mainly dependent on ubiquitination), forming complexes with STAM, Eps15, and Clathrin (Vechetti et al., 2021). HRS then recruits the tumor susceptibility gene TSG101 (a component of ESCRT-I), and ESCRT-0 and ESCRT-I are jointly responsible for sorting the ubiquitination protein. ESCRT-I is a heterotetramer composed of VPS23, VPS28, VPS37, and MVB12 (Pashkova and Piper, 2012). ESCRT-II: ESCRT-II is a Y-type heterotetramer protein complex composed of VPS36, VPS22, and VPS25. The N-terminal of VPS36 binds to ubiquitin (the cargo protein or other proteins on ESCRT) and the C-terminal of ESCRT-I. The supercomplex of ESCRT-I/II has been shown to play an important role in membrane deformation of ILVs. VPS25 of ESCRT-II and VPS20 of ESCRT-III have the ability to combine with high affinity, and when the two are combined, it initiates the work of ESCRT-III. ESCRT-III: In yeast, ESCRT-III consists of four core subunits: VPS20, Snf7 (VPS32), VPS24, and VPS2, as well as helper proteins Did2, VPS60, and Ist (Capalbo et al., 2012). These subunits are inactive monomers in the cytoplasm and form a transient ESCRT-III heteropolymer once combined on the membrane. Its main function is to promote membrane separation and allow it to enter the endosomal compartment in the form of ILVs to form MVBs (Kesidou et al., 2020).

## 2 The role of exosomes in virus infection

### 2.1 Relationship between exosomes and virus infection

Most cells release exosomes during normal growth. Exosomes are an important part of viral pathogenesis and immune response, and can carry intracellular substances to participate in intercellular information exchange, which also contributes to the transmission of viral nucleic acids and proteins between cells (Liu et al., 2016b). Many studies have shown that exosomes are the vectors of DNA and RNA viruses (As shown in **Table 1**: describes how RNA enters the exosomes). Viruses can regulate the intracellular environment of host cells by wrapping viral nucleic acid, viral protein, and even the whole virion in exosomes to help viruses proliferate and replicate, and make use of the transport function of the exosome to follow the circulation of body fluids to the target cells for diffusion and infection (Meckes and Raab-Traub, 2011). For example, studies of several RNA viruses have shown that exosomes summon viral proteins to integrate into exosomes (Angela et al., 2015). The exosomes isolated from the supernatant of HIV-infected cells contain Gag and Nef proteins (Fujinaga and Cary, 2020). Similarly, HTLV-1’s trans-activating protein Tax, which is an important amplification and conversion factor for T cells, can also be integrated into exosomes released by viral infection supernatant (Harendra et al., 2015). Exosomes secreted by Hepatitis C virus (HCV) infection contain not only HCV core proteins, but also apolipoprotein ApoE and ApoB (Bandopadhyay and Bharadwaj, 2020). In the serum of HCV infected patients, the E2 protein of the virus and CD81 can also co-locate (Favre and Muellhaupt, 2005). Exosomes have characteristics similar to some viruses, including biogenesis, molecular properties taken up by cells, and intercellular transfer of functional RNAs, mRNAs, and cellular proteins (Alenquer and Amorim, 2015). Differences that exist between exosomes and some viruses include the complexity of self-replication after infection with new cells, temporarily regulated virus expression, and the complexity of virus entry (Xu et al., 2020). Exosomes extracted after virus infection of cells can transmit many regulatory factors, which change cargo transport and cause the host to produce an effective immune response to pathogens, including activation of antiviral mechanisms and transfer of antiviral components in a variety of cells (Xu et al., 2022), and can detect the presence of pathogens that promote viral infection. Exosomes containing viral genomes can accelerate virus transmission by entering susceptible cells. At the same time, some specific contents of exosomes can also play an anti-viral role in antiviral infection by inhibiting viral replication or inducing an antiviral immune response (Li et al., 2019). These contents enter the recipient cells through exosome transmission and transport, and participate in intracellular communication and life activities.

**Table 1:** Different ways of RNA entering the exosomes.

Types of RNA	Different ways of entering the exosomes
mRNA	Preloading method: heavily dependent on the production cells in the biogenesis process to package mRNA goods into exosomes (Olanrewaju and Hakami, 2020).
	Active preloading method: The production cells were transfected with two types of plasmids (Hirata et al., 2018). A plasmid encodes a fusion protein (surface marker CD9, CD63, or cell membrane protein Hspa8) composed of mRNA-binding components and Exosomes-enriched proteins (Li Z. et al., 2022). The mRNA of interest transcribed from the plasmid contains deliberately designed recognition sites that can specifically bind to the mRNA of the fusion protein (Zhao et al., 2021). The rest of the fusion protein, namely Exosomes-enriched protein, is incorporated into exosomes in the process of biogenesis to realize the preloading of active mRNA (Yan et al., 2022). Another method is targeted and modular exosomes loading (TAMEL) (Yin et al., 2022a). By fusing exosomes-rich proteins into the coat protein of MS2 phage, mRNA is actively loaded into the exosomes (Yang et al., 2023). Then the homologous stem-ring sequence was integrated into the mRNA vector to promote mRNA binding and load into the exosomes (Li Z. et al., 2022).
	Post-loading method: Exogenous mRNA is loaded into isolated exosomes by electroporation or chemical transfection reagent (Liu et al., 2015). Electroporation is a commonly used method of loading various molecules, including siRNA and miRNA, as well as mRNA, into the purification of exosomes (Koh et al., 2023). Another commercial loading reagent called REG1 is also used to load mRNA into exosomes after its separation (Aslan et al., 2021).
miRNA	Blood cells and mononuclear lymphoma cells THP1 can actively and selectively package miRNA into exocrine bodies and secrete it into the body circulation in response to various stimuli (Badimon et al., 2017).
	Neutral sphingomyelinase 2 (nSMase2), which controls ceramide biosynthesis, can regulate the secretion of exosome miRNA (Nishida-Aoki and Ochiya, 2015). The increase of miRNA secretion mediated by nSMase2 increases the number of exosomes released by cells and the number of miRNA packaged into exosomes (Khalyfa and Gozal, 2014). NSMase2 is a key factor in determining the infiltration of miRNA into exosomes or RNA-binding proteins (Song et al., 2013).
	Specific protein control of miRNA entry into exosomes by RNA-induced silencing complex (RISC) (Li C. et al., 2021). By combining with AGO2, the GW182 required for miRNA function is enriched in exosomes (Chiou et al., 2018). RISC is involved in the process of packaging miRNA into exosomes (Shirazi et al., 2024).
lncRNA	LncARSR is regulated by hnRNAPA2B1 and packaged into exosomes (Jiang et al., 2023).
	HnRNPA2B1-mediated packaging of LNMAT2 into exosomes secreted by BCa cells (Tang et al., 2021).

### 2.2 The role of exosomal miRNAs in virus infection

**Figure 3:**
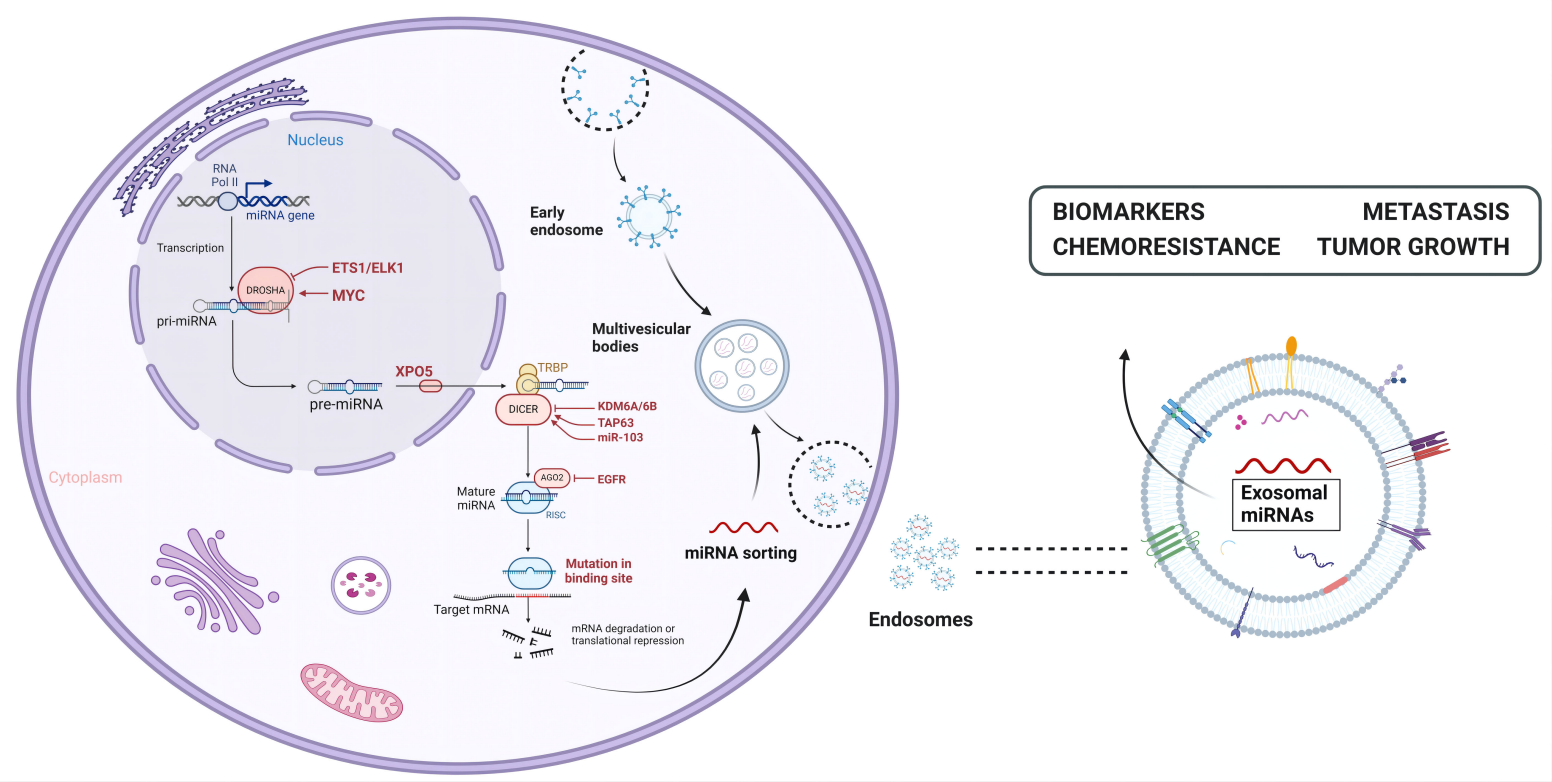
The biogenesis and functions of exosomal miRNAs. miRNAs are transcribed from RNA Pol II in the nucleus into primary mirnas (pri-miRNAs) and further processed and clipped into precursor RNA with a stem-ring structure (pre-miRNAs), which are then exported to the cytoplasm via exportin-5. In the cytoplasm, the 5 ‘end and 3’ end of the precursor RNAs are cut by Dicer enzyme to form mature miRNAs, which are then wrapped and released by exosomes to play their role.

Exosome miRNAs are regulatory non-coding RNAs, which are usually encapsulated into exosomes as signal molecules. They are about 19–25 nucleotides in length and play an important role in regulating gene expression, translation, and information transmission between cells; a single miRNA can target hundreds of mRNAs, and multiple miRNAs may regulate a single mRNA and affect the expression of many genes in the organism (Lee et al., 2021). The typical function of miRNAs is to regulate the stability of mRNA by identifying the 3’ untranslated region (3’ UTR) of mRNA, thus affecting the level of gene expression (Papadakos et al., 2023). miRNAs are often encapsulated by exosomes and protected from degradation (**Figure 3**). Therefore, the changes of exosomal miRNAs affect the degree of regulation of target genes, and then affect the homeostasis of the organism. miRNAs generally participates in a series of biological processes by cleaving target genes to degrade target genes and inhibiting protein translation (Pan et al., 2022). miRNAs are considered the most promising non-invasive biomarker because of their high abundance, good stability, and regulation effect on cells (Miyoshi et al., 2022). Understanding the role of exosomal miRNAs in the pathogenesis of infectious diseases and their therapeutic potential is necessary for the development of new therapeutic approaches. It has been proven that a variety of viruses can self-replicate and infect with the functional properties of exosomal miRNAs, such as: Human immunodeficiency virus (HIV), Hepatitis C virus (HCV), Enterovirus 71 (EV71), Epstein-Barr virus (EBV), Hepatitis B virus (HBV), and Severe acute respiratory syndrome coronavirus 2 (SARS-CoV-2), they are listed in **Table 2**.

**Table 2:** The function and mechanism of exosomal miRNAs secreted by different viruses.

Virus	Recipient exosomes	miRNA	Function	Mechanism of action	References
HIV	Serum exosomes	vmiR-TAR	Down-regulation of apoptosis prolongs the incubation period of the virus to produce more virus	Reduces the level of Cdk 9 and Bim and silences the mRNA of Bcl-2 interacting protein in receptor cells	(Martini et al., 2020)
		vmiR88, vmiR99	Promote the release of a large number of inflammatory cytokines	It can activate TLR8-mediated signaling pathway in macrophages	(Kaur and Kumar, 2021)
	Exosomes secreted by T-cell	miR-155–5p	Transfer directly from HIV-1-infected T cells to cervical cancer cells via exosomes and activate the NF-κB signaling pathway by reducing the expression of their target gene ARID2	Promote the infection of cervical cancer cells	(Güler et al., 2021)
	Plasma exosomes	miR-146a, miR-126, miR-21, miR-let-7a	Mediated immune regulation and neuroinflammation	The expression level is upregulated after HIV infection	(Khalid et al., 2022)
HCV	Exosomes secreted by Hepatocyte	miR-122	Anti-HCV antibodies are produced during HCV infection	Stimulates B cell proliferation and activation	(Yin et al., 2022b)
		let-7b, miR-206	As the ligand of TLR7	Induction of Macrophage production by exosomal delivery BAFF	(Liao et al., 2021)
	Exosomes secreted by umbilical cord mesenchymal stem cells	miR-145,miR-199a, miR-221	Largely contributed to the suppression of HCV RNA replication.	These miRNAs have binding sites on HCVRNA, have unique expression profiles, and are representative functional miRNAs	(Zhou L. et al., 2022)
		miR-4718, miR-642a-5p, miR-6826–3p, miR-762	Down-regulated expression induces cell proliferation and prevents apoptosis *in vitro*.	It was positively correlated with the expression pattern in liver	(Zhang et al., 2021)
EV71	Exosomes secreted by human oral epithelial cells	miR-30a	Inhibition of type I IFN reaction	It is delivered to macrophages and targeted by bone MyD88	(Hu et al., 2023)
	Exosomes secreted by THP-1	miR-146a	The concentration was significantly increased and could be transferred into target cells	Inhibition of type I interferon response and functional transfer to recipient cells	(Li et al., 2019)
EBV	Exosomes secreted by B-cell	hsa-miR-21–5p, hsa-miR-146a-5p	Regulation of host inflammatory response	Expression disorder in EBV infection	(Hatton et al., 2014)
	Exosomes in cerebrospinal fluid of RRMS patients	miR-BART9–3p, miR-BART15	It provides a substantial connection for the transformation from EBV infection to MS.	Expression increased in EBV infection	(Mohammadinasr et al., 2024)
	Exosomes secreted by LCL	miR-BART3	Implicated in the regulation of innate immunity	Targeted introduction of IPO7 induces pro-inflammatory cytokine IL-6	(Li J. et al., 2022)
		miR-BHRF1–1	Strongly potentiate the transforming properties of EBV	Down-regulation of host p53 protein in nasopharyngeal carcinoma	(Li J. et al., 2022)
HBV	Plasma exosomes	miRNA-1246, miRNA-150–5p, miRNA-5787, miRNA-8069,	It can be used as new markers of miRNAs of peripheral blood plasma exosomes after HBV infection	Combined diagnosis using multiple markers is more effective	(Wang D. et al., 2022)
	Serum exosomes	miRNA-574–5p	The expression of HBV polymerase was inhibited	By binding HBV genome sequence at 2750 ~ 2757 positions	(Khanam et al., 2021)
		miR-222–3p	Promote anti-apoptosis, proliferation and drug resistance of hepatocellular carcinoma cells	Induce the expression of miR-135a-5p and pass through the VAMP2 axis	(Wei et al., 2021)
		miR-125b	miR-125b was an independent predictor of HBV DNA, HBsAg, and HBeAg levels.	miR-125b was up-regulated in HBV patients	(Zhou et al., 2018)
		miR-146a	Enhanced HBV replication	Regulation of TLR4 inhibits M1 immune function	(Hou et al., 2016)
		miR-142–3p	Promote the proliferation, migration and invasion of liver cancer cells	SLC3A2 promotes iron death in HBV-infected M1-type macrophages and affects the production of GSH, MDA and Fe^2+^.	(Alenquer and Amorim, 2015)
		miR21, miR-192, miR-215, miR-221, miR-222	Lead to immune escape of HBV	Targeting the IL-21 gene decreases its expression	(Enomoto et al., 2017)
	Exosomes secreted by hepatocytes	miR-222	Promotes the activation of LX-2 cells	Inhibition of iron death in LX-2 cells induced by TFRC	(Liu et al., 2016a)
	Exosomes secreted by HepG2-NTCP	miR-21	Promotes cancer progression by activating cancer-associated fibroblasts (CAF)	Converting normal HSC into CAF by directly targeting PTEN leads to activation of PDK1/AKT signal in HSC and secretion of angiogenic cytokines, which can down-regulate IL-12p3 mrna expression	(Zhao et al., 2020)
		miR-29a	Causes obstruction of HBV clearance	The expression level of IL-12p40 gene A is down-regulated, the activity of IL-12 is decreased, and the proliferation and activation of NK and CTL cells are affected	(Chaudhari et al., 2022)
SARS-CoV-2	Serum exosomes	miR-223–3p, miR-24–3p, miR-145–5p, miR-75p	Inhibition of SARS-CoV-2 replication	It directly binds to S-protein-specific target sites and mediates membrane fusion and entry into SARS-CoV-2	(Mishra and Banerjea, 2021)
	Exosomes secreted by Activate Human Microglia	miR-148a	As an immunomodulator	Transported into human microglia and inhibited USP33 and downstream IRF9 protein expression levels	(Zhang et al., 2020)
		miR-590	Direct targeting of IRF9 expression levels	miR-590 binds strongly to the complementary IRF9 3’ UTR sequence	(Estep et al., 2020)
	MSC-derived exosomes	miR-125a-3p, miR-125b-1–3p	Minimizes cell death and works collaboratively to reduce inflammation throughout the body	It can be targeted to bind to the 3’ UTR region of multiple genes	(Wang H. et al., 2022)
		miR-769–3p, miR-202–3p	Reduce cell death and avoid tissue damage	Synergistically target the 3’ UTR region of the TNF e IFN gene that inhibits its protein translation	(Lei et al., 2022)
		miRNA let-7e-5p	The anti-apoptotic effect is mediated by the transfer of miRNA let-7e-5p	Binds to the 3’ UTR region involved in cell death signaling pathways	(Wu et al., 2020)

#### 2.2.1 Human immunodeficiency virus (HIV)

Exosomes secreted by HIV-infected cells and patient serum carry transactivation response element (TAR) RNA as well as its products vmiR-TAR, vmiR88 and vmiR99 (Martini et al., 2020). TAR RNA is located in HIV 5’ long terminal repeat (5’-LTR) with a 52-base RNA stem-loop structure. vmiR TAR reduces the level of cyclin-dependent kinase 9 (Cdk 9) and Bcl-2 interacting mediators of cell deaths (Bim) and silences the mRNA of Bcl-2 interacting protein in receptor cells (Kaur and Kumar, 2021). The down-regulation of apoptosis prolongs the incubation period of the virus to produce more viruses. vmiR88 and vmiR99 encoded by HIV 5’-LTR can activate the Toll-like receptor 8 (TLR 8)-mediated signaling pathway in macrophages, and promote the mass release of preinflammatory cytokines, such as tumor necrosis factor-α (TNF-α) (Güler et al., 2021). After HIV-1 enters the incubation period, due to the poor recognition of miRNA by the immune system, the virus takes advantage of exosomes to release miRNA, making neighboring cells more susceptible while accelerating viral infection (Khalid et al., 2022). HIV-infected T-cell-derived miR-155–5p is transferred directly from HIV-1-infected T cells to cervical cancer cells via exosomes while activating the NF-κB signaling pathway by reducing the expression of its target gene ARID2, thus promoting the invasion of cervical cancer cells (Li J. et al., 2022).

#### 2.2.2 Hepatitis C virus (HCV)

HCV-infected hepatocellular derived miRNAs (such as miR-122, let-7b and miR-206) as TLR7 ligands can induce macrophages to produce the B cell activating factor (BAFF) through exosomal delivery, while hepatocellular derived exosomal miR-122-induced BAFF can stimulate B cell proliferation and activation, meanwhile anti-HCV antibodies are produced during HCV infection (Yin et al., 2022b). Tlr3-activated macrophages impart anti-HCV activity to hepatocytes via exosomes containing members of the anti-HCV miRNA-29 family (Zhou L. et al., 2022). In addition, exosomes secreted by umbilical cord mesenchymal stem cells inhibit HCV infection by transferring multiple mirnas, including miR-145, miR-199a and miR-221 (Zhang et al., 2021). The expression patterns of miR-4718, miR-642a-5p, miR-6826–3p and miR-762 in exosomes were positively correlated with those in the liver, and the down-regulation of these miRNAs induces cell proliferation and prevents cell apoptosis *in vitro* (Cabral et al., 2022).

#### 2.2.3 Enterovirus 71 (EV71)

EV71 is a single plus-stranded RNA virus. The exosomes of EV71-infected RMS cells contain EV71 RNA, viral capsid protein VP1 and EV71 particles, which, partially resistant to antibody neutralization, can establish an effective infection *in vitro* (Gu et al., 2020). However, exosomes of human oral epithelial cells infected with EV71 selectively package high levels of miR-30a, which can be delivered to macrophages and inhibit the type I IFN response by targeting the bone marrow differentiation factor 88 (MyD88), thereby enhancing viral replication (Hu et al., 2023). It can induce the increase of exosomes secreted by colon cancer cell line HT29 and human mononuclear/macrophage cell line THP-1, meanwhile the EV71 virus nucleic acid carried in those exosomes can break through the limitation of virus-specific receptors and assemble its own genes into exosomes to expand the range of host infection (Li et al., 2019). After infection, the enrichment of secreted exosome miR-146a significantly increases and can be transferred into target cells, which can be functionally transferred to recipient cells by inhibiting the type I interferon response, while promoting the replication of exosome EV71 RNA in recipient cells (Fu et al., 2017).

#### 2.2.4 Epstein-Barr virus (EBV)

EBV expresses 44 mature miRNAs derived from 25 miRNA precursors encoded by 2 primary transcripts BamHI fragment H to the right open reading frame 1 CSF (BHRF1) and BamHI fragment A to the right transcription (BART), which are important for cell survival and proliferation during the pre-infection incubation period of B cells (Gallo et al., 2020). EBV miRNAs can regulate genes involved in apoptosis, antigen presentation and recognition as well as B-cell transformation (Hassani et al., 2019). Chronic infection can alter the expression of host miRNA, thereby regulating the host inflammatory response (Mourenza et al., 2022). The dysregulation of host miRNA can affect B cell function. miRNAs and EBV miRNAs of B cells are dysregulated before and after EBV infection, and participate in the pathogenesis of multiple sclerosis (MS) through interactions with MS risk sites (Afrasiabi et al., 2021). EBV miRNAs, including Bart miRNAs, have important functions in cancer growth, tumor invasion and host immune surveillance (De Re et al., 2020). Studies have found that EBV infection can induce the expression of some miRNAs in B cells, including hsa-miR-21–5p and hsa-miR-146a-5p, which participate in tumorigenesis and are dysregulated in EBV infection (Hatton et al., 2014). *In vitro*, BART miRNA can induce macrophages to produce immunomodulatory phenotypes (Song et al., 2017). It is characterized by the gene expression of interleukin 10 (IL10), TNF-α and arginase 1 (Arg1) (Jourdan et al., 2017). The elevated expression of miR-BART9–3p, miR-BART15 and inflammatory cytokines in the cerebrospinal fluid exosomes of patients with relapsing multiple sclerosis (RRMS) provides a substantial link between EBV activity and disease pathogenesis as well as the transition from EBV infection to MS (Mohammadinasr et al., 2024). The exosomes secreted by EBV-infected lymphoblastic cell line (LCL) contain miR-BART3 and miR-BHRF1–1, which are pathways for the transfer of viral miRNAs (Li J. et al., 2022). miR-BART3 targets the introduction of importin 7 (IPO7), induces the pro-inflammatory cytokine IL-6, and plays an anti-apoptotic role against caspase 3, and miR-BHRF1–1 enhances EBV replication by down-regulating host p53 in nasopharyngeal carcinoma.

#### 2.2.5 Hepatitis B virus (HBV)

Hepatitis B virus (HBV) is the pathogen of hepatitis B and its related diseases and is characterized by long-term chronic infection with hepatocyte damage and complex interaction between HBV and the immune system (Sahasrabuddhe et al., 2012). Studies have found that exosomal miRNAs from HBV-infected cells can inhibit the immune response and lead to the dysfunction of immune cells, thereby interfering with the clearance of HBV from host cells and resulting in persistent infection of the virus (Lin et al., 2023). After HBV infection, the expression levels of some miRNAs in plasma and serum exosomes changed significantly and participated in the process of virus infection to varying degrees. The function and mechanism of miRNAs in the exosomes during HBV infection are listed in **Table 2**. A large number of studies have demonstrated that exosomal miRNAs can be used to monitor the progress of HBV and as biomarkers for for early detection of hepatocellular carcinoma (HCC).

#### 2.2.6 Severe acute respiratory syndrome coronavirus 2 (SARS-CoV-2)

SARS-CoV-2 belongs to the coronavirus group and is a positive RNA single-stranded virus with potential pathogenicity associated with respiratory diseases. The genome has 14 open reading frames (ORF), encoding 27 proteins, 4 of which are structural proteins, namely the envelope protein (E), nucleocapsid protein (N), matrix protein (M), and spike protein (S). Fifteen non-structural proteins (NSP) within the ORF1a and ORF1b regions are located at the 5’ end of the genome, and the 3’ end of the genome contains sequences associated with 8 helper proteins and structural proteins (Jamalkhah et al., 2021). The SARS-CoV-2 genome is wrapped by N proteins, while M and E proteins are key proteins to ensure the assembly of virus particles, and S proteins provide receptor specificity for viruses entry into cells (Maddali et al., 2024).

The coronavirus spike protein (S protein) is the outermost “coronavirus” structural protein that mediates the entry of the coronavirus into host cells (Wan et al., 2020). miRNA inhibits SARS-CoV-2 replication by inhibiting the expression of the targeted spike protein (S protein). Circulating miRNA in exocrine has similar effects to endogenous miRNA and can be delivered to receptor cells to regulate multiple target genes or signal events (Wang et al., 2024). Studies have shown that increasing the expression level of antiviral circulating miRNA can enhance their inhibition of SARS-CoV-2 replication. miRNA contained in exosomes released by SARS-CoV-2 S protein-transfected cells are transported into human microglia and inhibit ubiquitin specific peptidase 33 (USP33) (These are summarized in **Table 2**). The cellular level of USP33 regulates the turnover time of IRF9 through deubiquitination, effectively regulates the main pro-inflammatory factors of TNF-α, NF-κB, and IFN-β, and plays a role as a protective factor in inflammation (Mishra and Banerjea, 2021). Serum-derived exosomes have the potential to be used as a diagnostic tool for the detection of SARS-CoV-2, as well as a messenger RNA (mRNA) transmission carrier, even in asymptomatic patients, which is the limitation of the current practice of diagnostic testing around the world (Zhang et al., 2020). These results reveal that SARS-CoV-2 has become an indirect way to mediate central nervous system injury through the over-activation of human microglia, which provides a theoretical basis for finding new treatments for the neuropathogenesis related to SARS-CoV-2.

### 2.3 The role of exosomal ESCRT pathway in virus infection

**Figure 4:**
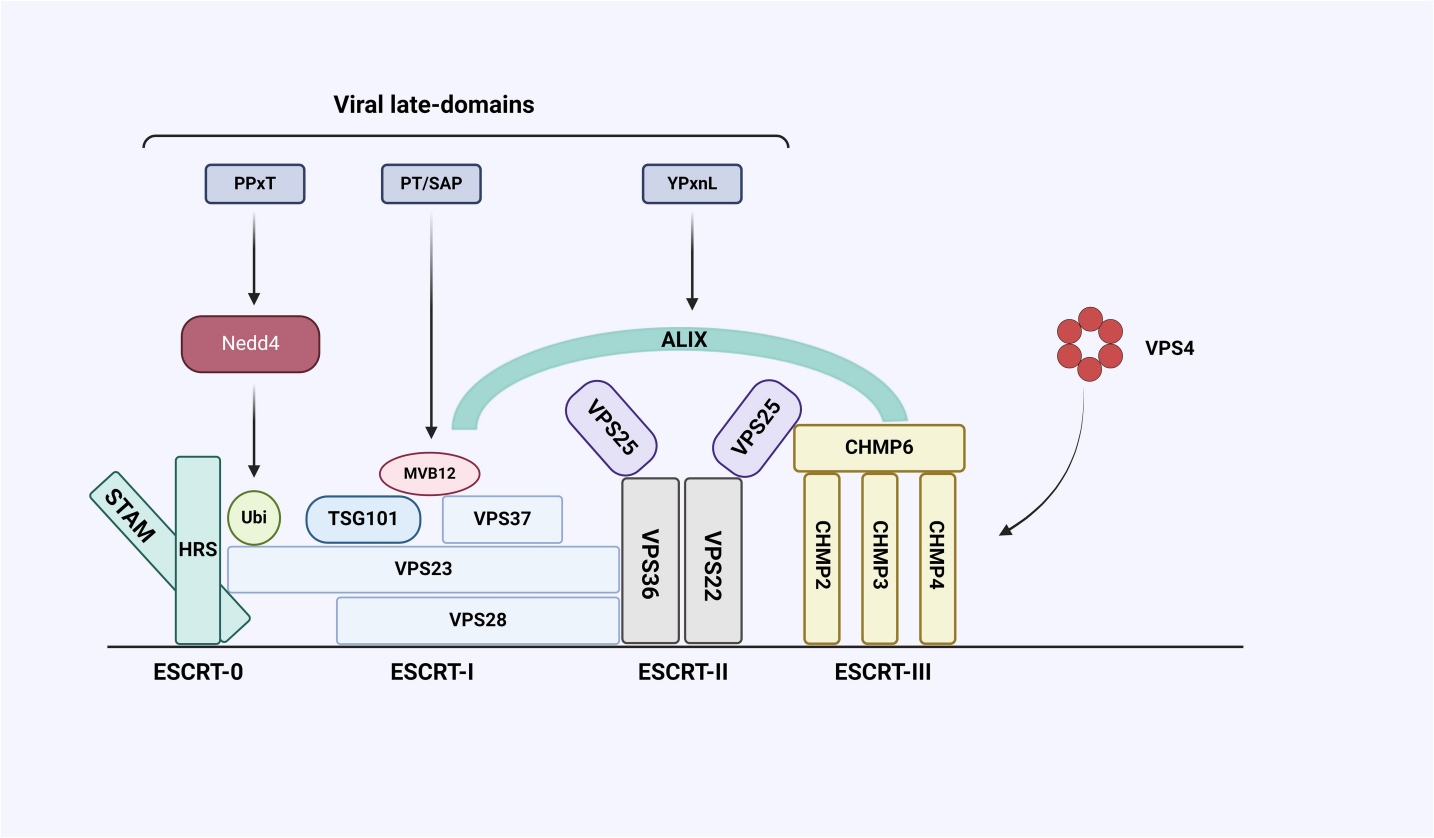
Interaction between ESCRT system and late-domain encoded by virus. The ESCRT system consists of five complexes, including ESCRT-0, I, II, III, and Vacuolar protein sorting-associated protein 4 (VPS4), and some auxiliary proteins including Alix. The viral structural protein Gag contains late-domains whose core sequences are PPxY, PT/SAP, and YPxnL. PPxY can bind to E3 ubiquitin ligase Neural precursor cell expressed developmental downregulated gene 4 (NEDD4), PT/SAP can bind to ESCRT-I subunit VPS23 and YPxnL can bind Alix.

Virus infection begins with the binding to the plasma membrane of the host cell, where the virus enters the host cell and is replicated and packaged, and then new virions leave the host cell and start a new infection cycle (Sharma et al., 2021). Many enveloped viral structural proteins include one or more short peptide sequences-late-domains, that facilitate the final separation of newborn viruses from infected cells, and the interactions between these domains and proteins involved in exosome formation are indispensable (Barton et al., 2016). So far, three types of domain motifs (PT/SAP, YXXL/YPXnL and PPxY) have been identified, and mutations or deletion of these short peptide sequences will lead to accumulation of immature virions on the plasma membrane (Urata and Yasuda, 2012). The enveloped RNA virus itself cannot synthesize most of the elements needed for budding, and its encoded Late-domains will hijack the host’s ESCRT pathway to complete virion budding (**Figure 4**). And studies have shown that retroviruses and many other viruses (Filoviruses, Arenavirus, Paramyxoviruses, Flaviviruses and Rhabdoviruses, etc.) use or require the ESCRT pathway for release (the following viruses are listed: Human immunodeficiency virus1 (HIV-1), Equine infectious anemia virus (EIAV), Herpes simplex virus types 1 (HSV-1), Ebola virus (EBOV)) (Igari et al., 2024), which participates in a variety of cellular functions, and its special feature acts from the inner surface of the bud to promote membrane budding and cut the membrane neck, but the specific mechanism of various proteins affecting virus production needs to be further studied (Little and Dwyer, 2021).

#### 2.3.1 Human immunodeficiency virus 1 (HIV-1)

Retroviral HIV-1 recruited ESCRT-I, ESCRT-III, and VPS4 to participate in its budding, which does not require ESCRT-0 and ESCRT-II in this process because the Gag protein of HIV-1 plays a similar role to ESCRT-0 and -PIN (Bigalke and Heldwein, 2017). The main HIV structure polyprotein Gag forms a polymer array under the plasma membrane, which leads to the change of membrane curvature. The PTAP and YPXnL motifs of Gag p6 domain are involved in exosomal biogenesis (Wang Y. et al., 2023). The PTAP sequence can bind the UEV domain of ESCRT-I protein TSG101 and mediate the recruitment of ESCRT-I complex to nuclear endosomes (Rose et al., 2020). The YPXnL motif located near the c-terminal of HIV-1 Gag protein can interact with the V domain in ALIX to recruit ESCRT-I and ALIX in the cell membrane region of virus assembly and budding, and then recruit ESCRT-III and VPS4 (Wang Y. et al., 2023) through ESCRT-I and ALIX protein, thus completing the whole process of virus budding. The final cutting involves the formation of ESCRT-II, which is caused by CHMP4B recruiting VPS4 through CHMP6 or ALIX (Meng et al., 2020). Early studies have confirmed the role of ESCRT-I and ESCRT-III in HIV budding (Moulin et al., 2023). However, it has not been proved until recently that ESCRT-II plays an indispensable role in membrane remodeling (Effantin et al., 2013). During HIV-1 infection, the elimination and deletion of ESCRT-II produced different effects. The elimination of ESCRT-II did not reduce the release of the virus, but the deletion of ESCRT-II had a similar effect as the deletion of ESCRT-I and ESCRT-III components, indicating that ESCRT-II is also necessary for effective budding of HIV (Effantin et al., 2013). In addition, ESCRT-I interacts with ESCRT-II and activates ESCRT-II through the C-terminal gall and H0 connecting domain of EAP45, and then recruits the CHMP6 of ESCRT-III to form an ESCRT-III-CHMP6 complex to function (Xia et al., 2023). HIV-1 Gag protein initiates virus assembly and budding, which requires ESCRT-III and VPS4 to isolate and release virions from the cell membrane, which is similar to the role of ESCRT-0 and ESCRT-II in MVB (Ju et al., 2021).

#### 2.3.2 Equine infectious anemia virus (EIAV)

EIAV is a special virus among the enveloped viruses studied so far. Due to the lack of TSG101/ESCRT-I binding sites, TSG101 does not participate in EIAV budding (Dowlatshahi et al., 2012). EIAV can only function with a new motif in Gag, YPDL, as a late-domain (Rivera-Cuevas et al., 2021). In contrast, YPDL is unique in that it can interact with the mu2 subunit of the AP-2 adapator protein complex and the ALIX protein respectively, which can only connect to the ESCRT system through ALIX, playing a key role in the release of virions in the late stage of EIAV budding (Ju et al., 2021). ALIX is involved in EIAV budding, responsible for attaching the YPDL of EIAV p9 Gag to host cell ESCRT-III (Dores et al., 2012). The N-terminal Bro1 domain of ALIX binds to CHMP4 and the central V domain binds to Gag protein (Sette et al., 2011). CHMP4B recruitment/polymerization helps control Gag polymerization and processing to ensure that ESCRT factor assembly and membrane fission occur at the appropriate stage of virion assembly (Sandrin and Sundquist, 2013). EIAV budding requires only a collection of ESCRT proteins, including ALIX, CHMP4B, CHMP2A and VPS4, which interact directly with each other (Sanford et al., 2014). In the process of finding new ways to prevent and treat EIAV, it is entirely possible to inhibit the interactions of these proteins or inhibit some of them to inhibit EIAV infection.

#### 2.3.3 Herpes simplex virus type 1 (HSV-1)

HSV-1 is a nervous system pathogen, which uses its own virus-encoded protein and host ESCRT mechanism to promote viral budding (Button et al., 2020). Unlike other enveloped viruses, HSV-1 does not require TSG101 or ALIX, a protein containing the Bro1 domain, to recruit or activate ESCRT-1 (Wang C. et al., 2023). HSV-1 capsid uses nucleoplasmic ESCRT to obtain a lipid envelope from the inner nuclear membrane (INM) and then fuses with the outer nuclear membranes (ONM) (Wilhelmsen et al., 2005). The herpesvirus nuclear exit complex (NEC) consists of viral proteins encoded by the UL31 and UL34 genes that induce perinuclear vesicles in uninfected cells (Draganova et al., 2021). In addition, HSV-1 NEC can mediate the budding of membrane vesicles in the absence of endogenous cellular proteins and ATP, where UL34 can interact with Alix in infected cells, and NEC recruits CHMP4 to the budding site of INM (Calistri et al., 2021). However, CHMP4/Vps4 controls the rate at which HSV-1 sprouts into the perinuclear space. When infected with HSV-1, ESCRT-III/Vps4 can promote the germination of HSV-1 from INM by playing a role in the biogenesis of autophagosomes, thereby degrading laminin (Butt et al., 2020). By recruiting ESCRT-III to the binding site of INM, it helps with HSV-bud while maintaining the integrity of INM (Calistri et al., 2021). Subsequent studies have shown that the secondary envelope of HSV-1 not only depends on VPS4, but also requires a functional ESCRT-III complex, and that the inactivation of any protein in ESCRT-III can effectively block the production of HSV-1 (Ju et al., 2021). ESCRT-III is the main driver of membrane remodeling and fracture containing a total of four core subunits, with the most abundant component being CHMP4, and it has been found that HSV-1 morphogenesis requires CHMP4C, but not CHMP4A or CHMP4B (Russell et al., 2020). At present, the specific mechanism of interactions between the HSV-1 structural protein and ESCRT components is still unknown to a large extent, which needs to be further studied.

#### 2.3.4 Ebola virus (EBOV)

EBOV is a single-stranded, enveloped RNA virus belonging to the Filoviridae family (Lv et al., 2024). EBOV virus matrix protein VP40 plays an important role in the later stage of virion assembly and release, recruiting ESCRT-1 complex proteins TSG101 and Alix proteins to the plasma membrane through vacuolar protein sorting (VPS) during budding (Silvestri et al., 2007). The L domain of VP40 mediates the separation of the virus from the host cell membrane by hijacking host proteins associated with the ESCRT pathway (Liang et al., 2017). VP40 contains two overlapping L-domain PPXY sequences and PT/SAP sequences capable of binding NEDD4 ubiquitin ligase, TSG101, and ALIX, respectively (Adu-Gyamfi et al., 2014). After TSG101 binds to EBOV VP40, normal functional sites are recruited from endosomes to the plasma membrane (Strickland et al., 2017). The entire ESCRT mechanism is recruited to the virus budding site. In the final sorting of the VPS pathway, the energy provided by the dissociated protein complex from VPS4 ATPase activity is required (Roshani et al., 2022).Studies have shown that VP40 can act independently of TSG101, directing each protein from nuclear weight to the cell surface (Okumura et al., 2008). Deactivation of VPS4 adenosine triphosphatase can reduce the budding rate by 80%, inhibition of VPS4 gene expression by phosphodiamine morpholino oligonucleotides can inhibit the toxicity of EBOV (Harty, 2009). These data results suggest that EBOV can use VPS protein budding to manipulate exosome tetraspanin proteins and ESCRT systems, up-regulate exosome biogenesis, and reveal VPS4 as a potential target for linear virus therapy (Zeng et al., 2022). In recent years, antiviral treatments targeting the interaction of TAP-TSG101 and PPXY-NEDD4 have been developed based on the research mechanism of ESCRT in EBOV infection, and it is believed that humans will be able to develop a treatment against EBOV in the future (Effantin et al., 2013).

## 3 Therapeutic potential of exosomes

Due to the biology of exosomes, they are able to reduce inflammation, cross the blood-brain barrier and have stability. Exosomes play an important role in the prognosis and diagnosis of a variety of pathological conditions such as cancers, neurodegenerative diseases, liver and kidney diseases as well as many cardiopulmonary diseases (Amiri et al., 2022). Recent studies have shown that exosomes are novel therapeutic agents for the treatment of cancers and other diseases (György et al., 2011). Msc-derived exosomes have the immunomodulatory and cytoprotective activities of parent cells, inhibit the expression of pro-inflammatory cytokines, exert the anti-inflammatory effect and promote tissue regeneration by enhancing extracellular matrix remodeling (Gurunathan et al., 2019). Exosomes can not only reproduce the biological potential of mesenchymal stem cells, but also have characteristics including targeted delivery, low immunogenicity and high repairability Bone marrow mesenchymal-stem-cell-derived exosomes (BM-MSC-Exos) have the anti-inflammatory, immunomodulatory and inhibitory effect on IFN-γ secreted by T cells (Lotfy et al., 2023). In addition, it also has advantages such as a low infection rate of pathogenic microorganisms, stable biological performance, low immune rejection after transplantation and high possible passage times, which is not easy to inactivate, but can interact with various types of cells (Raghav et al., 2021). Human adipose-derived stem cell (HASC) derived exosomes have better angiogenesis than BM-MSC-Exos, which not only promote angiogenesis, but also up-regulate early inflammatory responses, and can be used to improve graft rejection while inducing osteogenesis and adipogenesis, playing a key role in tissue repair and regeneration (Liu et al., 2022). In addition, it can inhibit cell apoptosis and regulate the immune system. Exosomes derived from human umbilical cord mesenchymal stem cells have a strong *in vitro* expansion and multidirectional differentiation ability, which can inhibit viral infection and replication, and promote the growth of new blood vessels, nerve regeneration as well as ossification of osteoblastic progenitor cells (Nasiry and Khalatbary, 2023).

The most representative application of exosomes in detection is undoubtedly the early diagnosis and disease monitoring of tumors (Dinish et al., 2014). Some liquid biopsy techniques are used to target exosomes. A large number of studies have found that exosomes derived from tumor cells contain a large number of specific miRNAs, and their biochemical properties are stable and easy to preserve, which can be used as markers for the early diagnosis of pancreatic cancer, colorectal cancer and other (Huang et al., 2022). Changes in the expression level of exosomal miRNA can reflect physiological and pathological changes while playing a regulatory role in the body, which is thus considered as a potential biomarker for diagnosis (Shufang and Ning, 2018). Tumor-specific circulating exosomal miRNAs have been developed as biomarkers for the early diagnosis of lung cancer. Exosomal miRNAs released by cancer cells can mediate phenotypic changes in TME cells, thereby promoting tumor growth and therapeutic resistance, such as fibroblast- and macrophage-induced differentiation (Wani et al., 2022). Cancer stem cells can transfer and enhance drug resistance in neighboring sensitive cancer cells by releasing exosomal miRNAs targeting anti-apoptotic and immunosuppressive pathways (Santos and Almeida, 2020). Exosomes induce resistance by carrying ABC transporters, which export chemotherapeutic drugs from recipient cells, thereby reducing drug concentrations to suboptimal levels (Colletti et al., 2021). Exosomal biogenic inhibitors represent a promising adjunctive therapeutic approach in cancer therapies, conferring drug resistance and survivability on tumor cells, and we still need to conduct in-depth research on this promising area (Das et al., 2020).

## 4 Conclusions

As nanoscale biological vesicles, exosomes safeguard their contents from degradation and facilitate intercellular transport, significantly influencing cellular pathophysiological processes, including immune defense, cell proliferation, and tumor metastasis. Viruses exploit the exosomal production mechanism to alter the host cell’s microenvironment, aiding their expansion and utilizing the humoral circulatory system to target and infect specific host cells, thereby evading the host’s immune response. The discovery of exosomal miRNAs offers fresh insights into the interactions between pathogens and immune cells, opens new avenues for immune regulation, and heralds innovative strategies for developing therapeutics against infectious diseases. However, numerous challenges remain. Advancements in research on exosomal miRNAs will unravel the pathogenic mechanisms of infectious diseases involving these miRNAs, pave the way for specific exosomal miRNA inhibitors, and explore their therapeutic potential. Future studies will aim to elucidate the action mechanisms and signaling pathways of exosomal miRNAs in treating infectious diseases, both *in vitro* and in animal models, to confirm their safety and efficacy. The involvement of the exosomal ESCRT system’s components in the budding and infection processes of viruses highlights the system’s role in viral proliferation and host cell invasion. Investigating the exosomal ESCRT pathway’s role in viral infections is crucial, presenting new prospects for developing targeted antiviral drugs and innovative vaccines.

Nonetheless, challenges persist in the practical application of exosomes, particularly concerning the safety and biocompatibility of exosomes from varied sources for drug transport. Enhancements in exosomal targeting techniques are imperative for clinical applications, and the rapid isolation, purification, and acquisition of clinically viable high-concentration exosomes remain critical hurdles to overcome. Advancements in technologies are essential to elucidate exosomal molecular properties and refine diagnostic and therapeutic approaches to align closely with clinical requirements. With robust support from proteomics, genomics, high-throughput sequencing, and bioinformatics data analysis, exosomes are poised to make significant strides in disease diagnosis, treatment, and clinical integration.

## Author contributions

HZ: Writing – original draft, Writing – review & editing. XL: Writing – review & editing. JS: Writing – review & editing. XS: Writing – review & editing. JX: Writing – review & editing. QM: Writing – review & editing. HD: Writing – review & editing.
